# Bcl-xL Genetic Modification Enhanced the Therapeutic Efficacy of Mesenchymal Stem Cell Transplantation in the Treatment of Heart Infarction

**DOI:** 10.1155/2015/176409

**Published:** 2015-05-05

**Authors:** Xiaodong Xue, Yu Liu, Jian Zhang, Tao Liu, Zhonglu Yang, Huishan Wang

**Affiliations:** ^1^Department of Cardiovascular Surgery, General Hospital of Shenyang Military Area Command, Shenyang Northern Hospital, Wenhua Road No. 83, Shenhe District, Shenyang 110016, China; ^2^Department of Cardiovascular Surgery, Xijing Hospital, Fourth Military Medical University, Xi'an, Shaanxi 710032, China

## Abstract

*Objectives.* Low survival rate of mesenchymal stem cells (MSCs) severely limited the therapeutic efficacy of cell therapy in the treatment of myocardial infarction (MI). Bcl-xL genetic modification might enhance MSC survival after transplantation. *Methods.* Adult rat bone marrow MSCs were modified with human Bcl-xL gene (hBcl-xL-MSCs) or empty vector (vector-MSCs). MSC apoptosis and paracrine secretions were characterized using flow cytometry, TUNEL, and ELISA *in vitro*. *In vivo*, randomized adult rats with MI received myocardial injections of one of the three reagents: hBcl-xL-MSCs, vector-MSCs, or culture medium. Histochemistry, TUNEL, and echocardiography were carried out to evaluate cell engraftment, apoptosis, angiogenesis, scar formation, and cardiac functional recovery. *Results*. *In vitro*, cell apoptosis decreased 43%, and vascular endothelial growth factor (VEGF), insulin-like growth factor-1 (IGF-1), and plate-derived growth factor (PDGF) increased 1.5-, 0.7-, and 1.2-fold, respectively, in hBcl-xL-MSCs versus wild type and vector-MSCs. *In vivo*, cell apoptosis decreased 40% and 26% in hBcl-xL-MSC group versus medium and vector-MSC group, respectively. Similar results were observed in cell engraftment, angiogenesis, scar formation, and cardiac functional recovery. *Conclusions.* Genetic modification of MSCs with hBcl-xL gene could be an intriguing strategy to improve the therapeutic efficacy of cell therapy in the treatment of heart infarction.

## 1. Introduction

Cell transplantation has emerged as a promising therapeutic approach for the restoration of heart function after myocardial infarction. Bone marrow mesenchymal stem cells (MSCs) are self-renewing, multipotent precursors of nonhematopoietic stromal tissues. Under appropriate conditions, MSCs can be induced to differentiate into multiple cell lines, which includes osteoblasts, chondrocytes, adipocytes [1], skeletal muscle cells [2], cardiomyocytes [3], hepatocytes [4], and neural cells [5]. MSCs were demonstrated to be able to promote angiogenesis and the survival of ischemic cardiomyocytes through the paracrine production of various cytokines [6, 7]. Furthermore, it was shown that MSCs are immunosuppressive favoring the inhibition of inflammatory responses and the future fibrosis of the injured heart tissue [8, 9]. MSCs can be easily isolated from the bone marrow or adipose tissue and expanded* in vitro* based on their ability to adhere to culture dishes. Therefore, MSCs appear to be an appealing cell source for transplantation therapy in myocardial infarction.

However, within the first few days after transplantation, the low survival rate of MSCs incurred from the deleterious microenvironment of ischemia, inflammatory response, and proapoptotic factors severely holds back the therapeutic effects on the cardiomyocytes restoration [3, 10]. Thus, it is necessary to reinforce MSCs to improve the efficacy of cell therapy. Evidences have demonstrated that genetic modification of MSCs with survival [11] or antiapoptotic [12] genes can improve the viability of the transplanted MSCs and results in a better homing of MSCs into the ischemic microenvironment, thus enhancing the cardiac functional recovery after acute myocardial infarction.

Bcl-xL and Bcl-2 are important regulators of cell apoptosis, both of which belong to the Bcl-2 protein family. It was demonstrated that the adenoviral mediated expression of human Bcl-xL (hBcl-xL) gene in rat heart can inhibit the apoptosis of ischemic cardiomyocytes after myocardial infarction and can prolong the cold preservation time period for cardiac transplants [13, 14]. And, also, it was reported that the overexpressed Bcl-2 gene can protect MSCs against apoptosis under hypoxic conditions both* in vitro* and* in vivo* and the transplantation of the Bcl-2 gene engineered MSCs may improve heart functional recovery after acute myocardial infarction [12]. Therefore, it is plausible to speculate that the overexpression of Bcl-xL gene in MSCs might also improve the viability of the transplanted MSCs and help to restore heart function after myocardial infarction.

In this study, we overexpressed hBcl-xL gene in adult rat bone marrow MSCs in order to enhance their survival against the deteriorative ischemic microenvironment after myocardial infarction. We proposed that genetic modification of MSCs with hBcl-xL gene could help to improve the viability of MSCs after transplantation into the ischemic heart and thereby lead to a better therapeutic effect on acute myocardial infarction.

## 2. Materials and Methods

### 2.1. Recombinant Lentivirus Construction

To construct the lentiviral expression vector pLenti6.3-IRES2-*EGFP*, the coding sequence of internal ribosome entry site- (IRES-) enhanced green fluorescent protein (EGFP) was inserted into the* Xho*I site downstream to the multiclonal site (MCS) region of pLenti6.3 CMV/V5 DEST (Invitrogen, Carlsbad, CA, USA). To generate the expression vector pLenti6.3-*hBcl-xL*-IRES2-*EGFP*, the 703 bp PCR product of hBcl-xL cDNA flanked with an* Asc*I and a* Pme*I restriction site at the 5′ and the 3′ end, respectively, was inserted in frame into the equivalently cut vector pLenti6.3-IRES2-*EGFP* under the control of the human cytomegalovirus (CMV) promoter. A Kozak consensus translation initiation site was added immediately before the start codon of hBcl-xL coding sequence to further increase the translation efficiency in eucaryotic cells. In this vector, the coding region for IRES could lead to the individual expression of EGFP together with the expression of hBcl-xL in the transduced cells.

### 2.2. Cell Transduction

Sprague-Dawley (SD) rat bone marrow MSCs (Cyagen Biotechnology, Guangzhou, China) were seeded into 6-well plates and cultured in low glucose Dulbecco's modified eagle medium (DMEM-LG) (Gibco, Grand Isle, NY, USA) supplemented with 10% fetal bovine serum (Gibco, Mulgrave, VIC, Australia), 100 U/mL penicillin, and 100 mg/mL streptomycin. When the cells were 50% confluent, the recombinant lentiviral construction pLenti6.3-*hBcl-xL*-IRES2-*EGFP* and the empty vector pLenti6.3-IRES2-*EGFP* were separately added into the culture medium at MOI = 50. Transductions were carried out for 24 h at 37°C; then the virus-containing medium was aspirated and the cells were cultured in fresh medium. Stably transduced cells were selected by adding 1 *μ*g/mL Blasticidin (Calbiochem, Billerica, MA, USA) into the culture medium.

### 2.3. Cell Imaging

5 × 10^5^ cells from each group of wild type MSCs, vector-MSC, and hBcl-xL-MSC were seeded into 6-well plate and cultured at 37°C for 24 hours. Cells were examined under an IX81 inverted microscope (Olympus, Tokyo, Japan) and photos were captured using a DP73 CCD digital camera (Olympus, Tokyo, Japan).

### 2.4. Western Blot Analysis

Cells were treated with RIPA lysis buffer (50 mM Tris-HCl (pH 7.4), 150 mM NaCl, 1% Triton X-100, 1% sodium deoxycholate, 0.1% sodium dodecyl sulfate, 2 mM sodium pyrophosphate, 25 mM *β*-glycerophosphate, 1 mM EDTA, 1 mM Na_3_VO_4_, and 0.5 *μ*g/mL leupeptin). The protein concentration of the samples was determined using Pierce BCA Protein Assay Kit (Thermo Scientific, Rockford, IL, USA). Proteins were resolved by 10–13% sodium dodecyl sulfate polyacrylamide gel electrophoresis (SDS-PAGE) and transferred to 0.22 *μ*m polyvinylidene difluoride (PVDF) membrane (EMD Millipore, Billerica, MA, USA) and probed with the first antibodies at 4°C overnight and then washed and incubated with horseradish peroxidase conjugated goat anti-rabbit IgG (Santa Cruz Biotech, Dallas, TX, USA) for 1 hour at room temperature. Reactive bands were developed and enhanced by SuperSignal West Pico chemiluminescence detection reagents according to the instructions of the manufacturer (Thermo Scientific, Rockford, IL, USA). The first antibodies used were polyclonal rabbit anti-Bcl-xL antibody (Cell Signaling Tech, Boston, MA, USA), polyclonal rabbit anti-cleaved caspase-3 antibody (Cell Signaling Tech, Boston, MA, USA), polyclonal rabbit anti-GAPDH antibody (Bioworld Tech, St. Louis Park, MN, USA) (loading control), and polyclonal rabbit anti-*β*-actin antibody (Bioworld Tech, St. Louis Park, MN, USA) (loading control).

### 2.5. Immunophenotypic Characterization

Cells were rinsed twice with PBS, trypsinized, and centrifuged at 200 ×g for 5 min and then resuspended in 500 mL PBS. Approximately 5 × 10^5^ cells per 100 mL were labeled with primary mouse antibodies against rat CD29, CD90, CD44, CD34, and CD45 at 4°C for 30 min and washed. The labeled cells were analyzed with a BD FASAria Cell Sorter (Beckton Dickinson, San Jose, CA, USA). The antibodies used in this experiment were CD29-FITC, CD90-FITC, CD44-FITC, CD34-FITC, and CD45-FITC (Beckton Dickinson, San Jose, CA, USA). Mouse IgG1-FITC (Beckton Dickinson, San Jose, CA, USA) was used as an isotype control.

### 2.6. Annexin V/Propidium Iodide (PI) Flow Cytometry Assay

1 × 10^6^ cells from each group of wild type MSCs, vector-MSC, and hBcl-xL-MSC were seeded into T25 flasks and were cultured in medium with 200 *μ*M PERDROGEN (H_2_O_2_) (Sigma, Santa Clara, CA, USA) at 37°C for 4 hours. After H_2_O_2_ treatment, Annexin V/PI apoptosis assay was performed using Annexin V-FITC Apoptosis Detection Kit I (Beckton Dickinson, San Jose, CA, USA). Briefly, cells were collected and washed in phosphate buffered saline (PBS) for two times and then resuspended in 500 *μ*L Binding Buffer. The resuspended cells were incubated with Annexin V-FITC and PI for 15 min and checked with a BD FASAria Cell Sorter (Beckton Dickinson, San Jose, CA, USA).

### 2.7. Enzyme-Linked Immunosorbent Assay (ELISA)

MSCs were cultured in a normoxic condition of 95% air and 5% carbon dioxide or a hypoxic condition of 95% nitrogen and 5% oxygen at 37°C for 24 hours. To assess the secretion of vascular endothelial growth factor (VEGF), insulin-like growth factor-1 (IGF-1), and plate-derived growth factor (PDGF), the conditioned medium was collected from wild type MSCs, vector-MSCs, and hBcl-xL-MSCs, respectively, and ELISA was performed using the VEGF Rat ELISA Kit (Abcam, Cambridge, Cambs, UK), PDGF-AA Rat ELISA Kit (Abcam, Cambridge, Cambs, UK), and IGF-1 Rat ELISA Kit (Abnova, Taibei, Taiwan) according to the manufacturers' protocol. The optic density (OD) value of each sample was read on an ELX-800 absorbance microplate reader (Biotek, Winooski, VT, USA) and adjusted with the TMB empty control. The concentration of each sample was calculated according to the standard curve.

### 2.8. Myocardial Infarction and MSC Transplantation

All animals were treated according to the Guide for the Care and Use of Laboratory Animals published by the US National Institutes of Health (NIH publication number 85-23, revised 1996) and all animal protocols were approved by the Animal Care and Use Committee of the General Hospital of Shenyang Military Area Command, Shenyang, China. Male SD rats weighing 280 g to 300 g were divided into three groups (medium group, vector-MSC group, and hBcl-xL-MSC group). Rats were anesthetized by intraperitoneal injection with pentobarbital (50 mg/kg), intubated via an endotracheal cannula, and mechanically ventilated. A left lateral thoracotomy was performed. The proximal portion of LAD artery was ligated with a 6-0 Prolene (Ethicon, Somerville, NJ, USA) suture. Immediately after ligation, a pale area was observed in the front wall of the left ventricle (LV) indicating a successful infarction. Following the ligation, animals received six subepicardial injections of vector-MSCs or hBcl-xL-MSCs around the infarction region. For each injection, 1 × 10^6^ cells were suspended in 50 *μ*L culture medium and injected into the 1.0 mm wide border region surrounding the infarcted heart muscle (considered to be border zone) with a 31-gauge needle (Beckton Dickinson, San Jose, CA, USA). The medium group received injections of culture medium into the same area. The sham group received a left lateral thoracotomy without LAD ligation. Intramuscular penicillin G benzathine (100,000 U/kg) was used to prevent infection.

### 2.9. Determination of Cell Engraftment

One or four weeks after MSC transplantation, animals were sacrificed and the hearts were dissected. From each heart, five frozen sections were prepared crossing the midlevel of the infarcted area. The sections were all fixed in ice-cold acetone and washed in PBS. Heart sections made at 1 week after transplantation were directly mounted with ProLong Gold antifade reagent with 4,6-diamino-2-phenylindole (DAPI) (Invitrogen, Carlsbad, CA, USA) and the heart sections from the later stage were stained with polyclonal rabbit anti-Troponin T (TnT) antibody (Cell Signaling Tech, Boston, MA, USA) and Cy3 conjugated goat anti-rabbit IgG (Santa Cruz Biotech, Dallas, TX, USA) and then mounted with ProLong Gold antifade reagent with DAPI (Invitrogen, Carlsbad, CA, USA). All sections were observed under a FV1000S-SIM/IX81 confocal microscope (Olympus, Tokyo, Japan). For each section, 10 high-power fields (HPF 400x) within the border zone were randomly selected and digitally photographed. Quantitative analysis of cell engraftment was performed with cellSens Entry software (Olympus, Tokyo, Japan). The integral optical density (IOD) of EGFP signal and total cell number in each microscopic field were calculated and cell engraftment was presented as the ratio of IOD and total cell number. An investigator blinded to the treatment performed the analysis.

### 2.10. *In Vivo* TUNEL Assay

Four weeks after MSC transplantation, animals were sacrificed and the hearts were dissected. Frozen sections were prepared from each heart crossing the midlevel of the infarcted region. TUNEL assay was performed as mentioned above and cell nuclei were counterstained with hematoxylin. For each slide, color images of 10 randomly chosen high-power fields (HPF 200x) within the border zone were captured and digitized under a BX53 microscope with a DP73 CCD digital camera (Olympus, Tokyo, Japan). Images were analyzed by an investigator who was blinded with respect to the MSC treatment. TUNEL-positive cells were defined as cells with clear brown-colored nuclear labeling. On each image, the total area of the brown-colored nuclei (area_brown_) and the area of total cell nuclei (area_total_) were calculated. The apoptotic index was represented as the ratio of area_brown_ and area_total_.

### 2.11. Evaluation of Capillary Density

For quantification of capillary density, 4 weeks after MSC transplantation, frozen sections were made from dissected hearts as mentioned above and stained with polyclonal rabbit anti-von Willebrand factor (vWF) antibody (Santa Cruz Biotech, Dallas, TX, USA) and horseradish peroxidase conjugated goat anti-rabbit IgG (Santa Cruz Biotech, Dallas, TEX, USA). Cell nuclei were counterstained with hematoxylin. Microscopic pictures were captured under a BX53 microscope with a DP73 CCD digital camera (Olympus, Tokyo, Japan). Capillary density was analyzed by an investigator who was blinded with respect to the MSC treatment. Positively stained capillaries were counted in 10 randomly chosen high-power fields (HPF 400x) within the border zone in 5 sections per animal. And capillary density was presented as the average number of vessels per high-power field.

### 2.12. Evaluation of Infarction Size

Four weeks after MSC transplantation, animals were sacrificed and the hearts were removed. Frozen sections were prepared as mentioned above and stained with Trichrome-Masson method. In this method, the collagen fibers were stained in blue color while the cardiac muscle fibers were shown to be red and the cell nucleus was black-blue. Scar formation was assessed by the quantitative analysis of collagen deposition. Image acquisition was performed under a BX53 microscope with a DP73 CCD digital camera (Olympus, Tokyo, Japan). Three continuous cross sections on the central level of the scar from each heart were selected and the collagen deposition was quantified using cellSens Entry software (Olympus, Tokyo, Japan). Scar size was presented as the percentage of the collagen deposition area to the whole section area of the left ventricle. An investigator blinded to the treatment performed the analysis.

### 2.13. Echocardiography

Four weeks after MSC transplantation, transthoracic echocardiographic studies were performed on the anesthetized animals. Left ventricle dimension and function were assessed using a 12 MHz high frequency liner phased-array transducer (Philips SONOS 5500, Bothell, WA, USA) by a blinded investigator. Left ventricular end diastolic dimension (LVDd) and systolic dimensions (LVDs) were derived from two dimensionally targeted M-mode tracings obtained along the parasternal short-axis view of the left ventricle at the papillary muscle level. Left ventricular ejection fraction (LVEF) and left ventricular fractional shortening (LVFS) were calculated. All measurements were performed and averaged over three consecutive cardiac cycles.

### 2.14. Statistical Analysis

Data were analyzed using SPSS version 12.0 for Windows (SPSS, Chicago, IL, USA). All values were presented as mean ± standard deviation (SD). One-way analysis of variance with Tukey's post hoc test was used to compare numeric data among the three experimental groups. Datasets consisting of two groups were compared with Student's *t*-tests. A level of *P* < 0.05 was considered statistically significant.

## 3. Results

### 3.1. EGFP and hBcl-xL Expressed in Genetically Modified Rat MSCs

**Figure 1:**
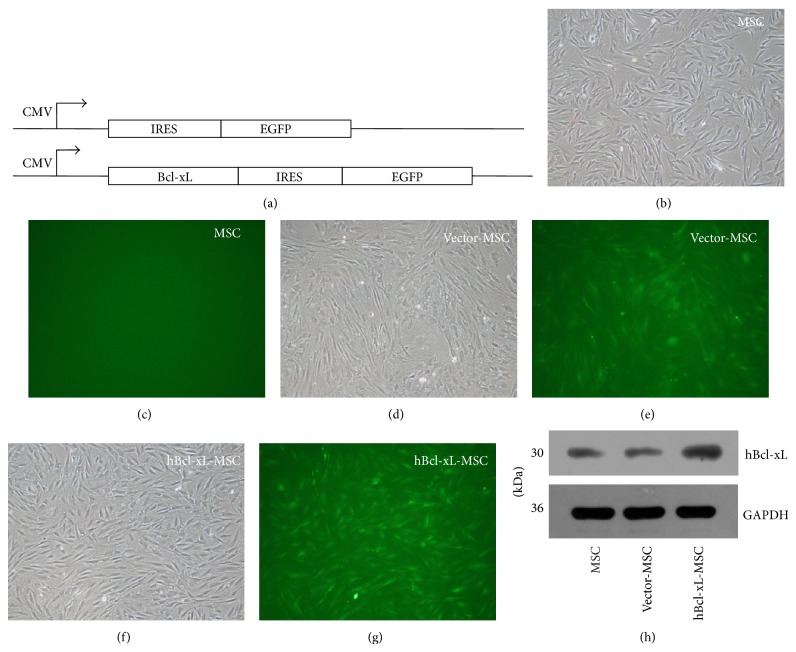
Expressions of EGFP and hBcl-xL in rat bone marrow MSCs. (a) Schematic representation of the coding regions of the viral vectors pLenti6.3-IRES-EGFP and pLenti6.3-hBcl-xL-IRES-EGFP. In vector pLenti6.3-hBcl-xL-IRES-EGFP, the insertion of IRES could lead to the individual expression of EGFP together with the expression of hBcl-xL in the transduced cells. All expressions were driven by CMV promoter. ((b)–(g)) Expressions of EGFP in wild type MSCs ((b) and (c)), vector-MSCs ((d) and (e)), and hBcl-xL-MSCs ((f) and (g)). (h) Western blot analysis of the expressions of hBcl-xL in MSCs, vector-MSCs, and hBcl-xL-MSCs. Data are representative of three independent experiments.

For the tracing of the genetically modified MSCs, we constructed expression vectors pLenti6.3-IRES2-*EGFP* and pLenti6.3-*hBcl-xL*-IRES2-*EGFP* (Figure 1(a)), where the individual expression of EGFP served as a tracking marker for the modified MSCs. To evaluate the expression of EGFP, wild type MSCs, vector-MSCs, and hBcl-xL-MSCs were seeded in a 6-well plate and cultured for 24 hours. Strong signals of EGFP were detected in vector-MSCs and hBcl-xL-MSCs while no signal was detected in wild type MSCs (Figures 1(b)–1(g)). Western blot analysis showed that the expression level of hBcl-xL in hBcl-xL-MSCs was remarkably higher than those in vector-MSCs and wild type MSCs (Figure 1(h)), which indicates the low level of endogenous expression of hBcl-xL in MSCs. Our results showed that both hBcl-xL-MSCs and vector-MSCs were successfully marked with EGFP, and hBcl-xL-MSCs were successfully modified with hBcl-xL.

### 3.2. Immunophenotypic Characterization of Genetically Modified Rat MSCs

**Figure 2:**
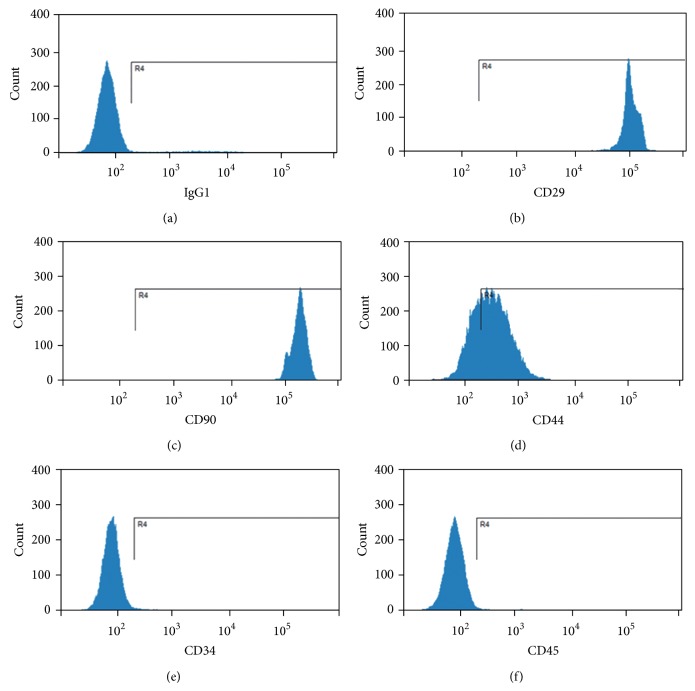
The surface marker expression of genetically modified rat MSCs. The expressions of selected surface markers of the modified MSCs were analyzed by flow cytometry. Mouse IgG1 was used as an isotype control (a); in the genetically modified MSCs, CD29, CD90, and CD44 were highly expressed ((b)–(d)) while the markers for hematopoietic stem cells, CD34 and CD45, were not expressed ((e) and (f)).

The surface marker expressions of the genetically modified bone marrow MSCs were identified by FCM analysis. It was shown that CD29, CD90, and CD44 were highly expressed in MSCs (Figures 2(b)–2(d)) while the markers for hematopoietic stem cells, CD34 (Figure 2(e)) and CD45 (Figure 2(f)), were not expressed. These expression patterns of the surface markers were similar to those of the primary cultured MSCs (see Supplementary Figure 1 in Supplementary Material available online at http://dx.doi.org/10.1155/2015/176409). This indicates that the genetic manipulation in this study did not alter the cell fate of the modified bone marrow mesenchymal stem cells.

### 3.3. Bcl-xL Modification Protected MSCs against Apoptosis* In Vitro*

**Figure 3:**
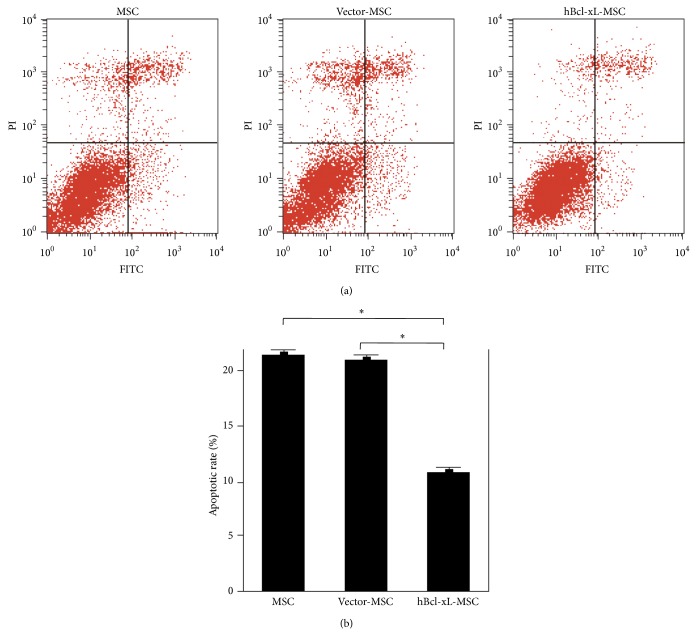
Antiapoptotic effect of hBcl-xL-MSCs* in vitro*. (a) Annexin V-FITC/PI apoptosisassay of wild type MSCs, vector-MSCs, and hBcl-xL-MSCs. Cells were seeded into T25 flasks and were cultured in medium with 200 *μ*M H_2_O_2_ at 37°C for 4 hours. The resuspended cells were incubated with Annexin V-FITC and PI for 15 min and checked with a BD FASAria Cell Sorter. (b) The apoptotic rate was presented as mean ± SD (*n* = 3, ^∗^ *P* < 0.05). PI: propidium iodide.

To examine the antiapoptotic ability of hBcl-xL modified MSCs* in vitro*, wild type MSCs, vector-MSCs, and hBcl-xL-MSCs were treated with 200 *μ*M H_2_O_2_ for 4 hours. Annexin V-FITC/(propidium iodide) PI apoptosisassay was carried out to evaluate cell apoptosis (Figures 3(a) and 3(b)). The results showed that the apoptotic rate of hBcl-xL-MSCs was significantly lower than those of wild type MSCs and vector-MSCs (10% ± 0.82% versus 21% ± 0.37% and 21% ± 0.13%, resp., *n* = 3, *P* < 0.05). These results demonstrated that hBcl-xL modification could protect MSCs against apoptosis under hypoxic conditions. We expected that hBcl-xL modification could also protect MSCs from apoptosis after transplantation into the infarcted myocardium.

### 3.4. Bcl-xL Modification Upregulated Angiogenic Cytokines in MSCs

**Figure 4:**
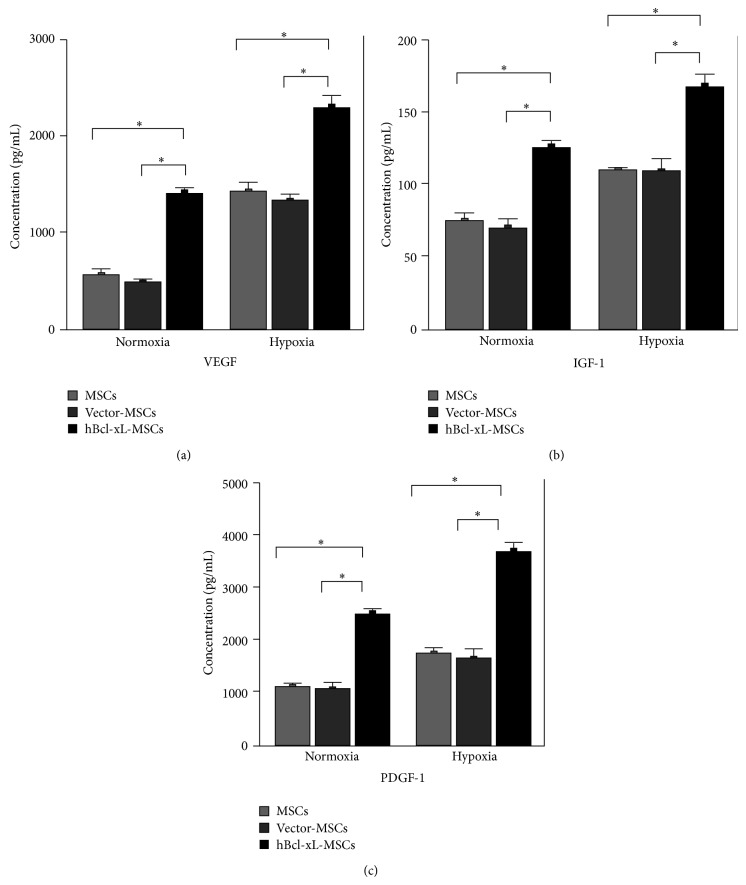
Upregulation of angiogenic cytokines in hBcl-xL-MSCs. ((a)–(c)) Evaluation of the paracrine secretion of VEGF (a), IGF-1 (b), and PDGF (c) by wild type MSCs (pale grey bar), vector-MSCs (dark grey bar), and hBcl-xL-MSCs (black bar). Cells were cultured under either normoxic or hypoxic conditions for 24 hours and the conditioned medium was collected for ELISA. Concentration values are mean ± SD (*n* = 3, ^∗^ *P* < 0.001). VEGF: vascular endothelial growth factor; IGF-1: insulin-like growth factor-1; PDGF: plate-derived growth factor.

Angiogenesis is a potential mechanism responsible for the therapeutic effect of MSC transplantation. We examined the MSC paracrine secretion of angiogenic cytokines VEGF, IGF-1, and PDGF. Wild type MSCs, vector-MSCs, and hBcl-xL-MSCs were cultured under either normoxic or hypoxic conditions for 24 hours. Cytokine secretions were examined by enzyme-linked immunosorbent assay (ELISA). The results showed that, under normoxic condition, the secretions of VEGF, IGF-1, and PDGF from hBcl-xL-MSCs were all significantly increased compared with wild type MSCs and vector-MSCs (increment: 1.5–1.9-fold for VEGF, 0.7-0.8-fold for IGF-1, and 1.2-1.3-fold for PDGF, *n* = 3, *P* < 0.05) (Figures 4(a)–4(c), normoxic). In response to hypoxia, angiogenic cytokine secretions were upregulated in all the three groups. The hypoxia-induced cytokine secretions of hBcl-xL-MSCs were much higher than those from wild type MSCs and vector-MSCs (increment: 0.6-0.7-fold for VEGF, 0.5-fold for IGF-1, and 1.1–1.3-fold for PDGF, *n* = 3, *P* < 0.05) (Figures 4(a)–4(c), hypoxic). These results suggested that hBcl-xL genetic modification could substantially enhance the angiogenic ability of MSCs, which may improve angiogenesis in the ischemic myocardium after MSC transplantation.

### 3.5. Bcl-xL Modification Increased the Engraftment of MSCs into Ischemic Myocardium

**Figure 5:**
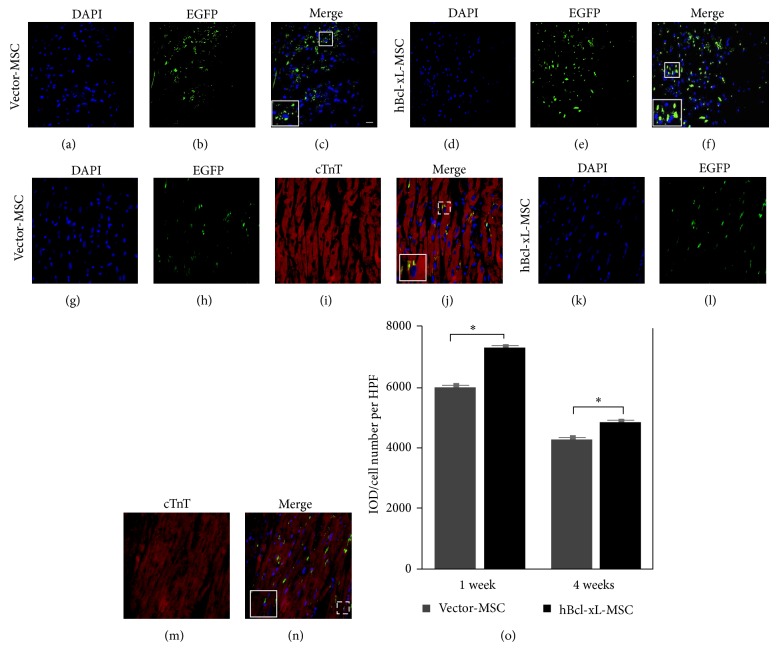
Engraftment of transplanted MSCs into infarcted myocardium. ((a)–(n)) Evaluation of MSC engraftment into the ischemic heart muscle at 1 week ((a)–(f)) and 4 weeks ((g)–(n)) after vector-MSCs ((a)–(c) and (g)–(j)) or hBcl-xL-MSCs ((d)–(f) and (k)–(n)) transplantation. Cell nuclei were stained with DAPI ((a), (d), (g), and (k)). The grafted MSCs were detected by EGFP signals ((b), (e), (h), and (l)) which appeared as irregular patches due to the cytoplasmic distribution of EGFP. The expanded squared areas in (c), (f), (j), and (n) indicate the MSC engraftments where the EGFP signals colocalized with DAPI. Myocardium was showed by Troponin T staining 4 weeks after cell injection ((i) and (m)). (o) MSC engraftment in vector-MSC group (dark grey bar) and hBcl-xL-MSC group (black bar) was presented as the ratio of IOD and total cell number per HPF. Values are mean ± SD (*n* = 6, ^∗^ *P* < 0.01). EGFP: enhanced green fluorescent protein; DAPI: 4,6-diamino-2-phenyl indole; IOD: integral optical density; HPF: high-power field. Scale bar represents 50 *μ*m (c) and 30 *μ*m (j), respectively.

To evaluate the engraftment of MSCs into the ischemic heart muscle after transplantation, rat left anterior descending artery ligation models were established. Immediately after ligation, for each animal, totally, 6 × 10^6^ vector-MSCs or hBcl-xL-MSCs in culture medium were injected into the border zone of the infarcted myocardium. The medium group received injections of culture medium into the same area. The evaluation of MSC engraftment was carried out at 1 week and 4 weeks after cell transplantation. Frozen sections were made from the dissected hearts crossing the midlevel of the infarcted area. Figures 5(a)–5(n) showed representative images of the grafted MSCs in the hearts of rats sacrificed 1 week (Figures 5(a)–5(f)) or 4 weeks (Figures 5(g)–5(n)) following vector-MSCs (Figures 5(a)–5(c) and 5(g)–5(j)) or hBcl-xL-MSCs (Figures 5(d)–5(f) and 5(k)–5(n)) transplantation. Cell nuclei were stained with DAPI (Figures 5(a), 5(d), 5(g), and 5(k)). The grafted MSCs were detected by EGFP signals (Figures 5(b), 5(e), 5(h), and 5(l)). Because of the cytoplasmic distribution of EGFP, it appeared as irregular patches that spread around or colocalized with the DAPI signals (squared areas in Figures 5(c), 5(f), 5(j), and 5(n)). To show the myocardial engraftment of MSCs, the heart sections prepared 4 weeks after cell injection were also stained with anti-Troponin T (TnT) antibody, a marker for cardiomyocyte (Figures 5(i) and 5(m)). In each microscopic field, integral optical density (IOD) of EGFP signal was calculated. MSC engraftment was represented as the ratio of IOD and the total cell number (Figure 5(o)). The results showed that, at both 1 week and 4 weeks after cell transplantation, the number of grafted MSCs in the hBcl-xL-MSC group was significantly increased comparing with the vector-MSC group (increment: 21.3% at 1 week and 13.2% at 4 weeks, *n* = 6, *P* < 0.01) (Figure 5(o)). These results suggested that hBcl-xL genetic modification could significantly increase the engraftment of MSCs after transplantation.

### 3.6. Bcl-xL Modification Protected MSCs against Apoptosis* In Vivo*

**Figure 6:**
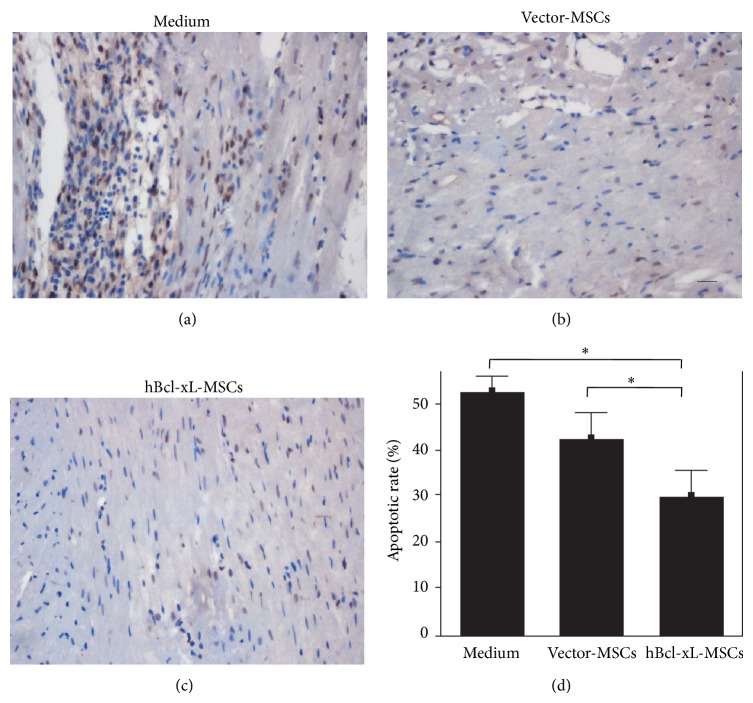
Antiapoptotic effect of hBcl-xL-MSCs* in vivo*. ((a)–(c)) TUNEL assay was performed at 4 weeks after transplantation of medium (a), vector-MSCs (b), or hBcl-xL-MSCs (c). TUNEL-positive cells were defined as cells with clear brown-colored nuclear labeling. (d) The apoptotic rate was presented as the ratio of the area of TUNEL-positive cell nuclei and the area of total cell nuclei. Values are mean ± SD (*n* = 6, ^∗^ *P* < 0.001). TUNEL: terminal deoxynucleotidyl transferase-mediated dUTP end labeling. Scale bar represents 50 *μ*m.

The increase in MSC engraftment into the ischemic myocardium suggested an antiapoptotic effect of hBcl-xL modification of MSCs* in vivo*. To assess the cellular protective effects of hBcl-xL modification of MSCs, 4 weeks after cell transplantation, animals were sacrificed and heart frozen sections were prepared as mentioned above. TUNEL assay was performed and the results showed that, in the border zone of the infarcted myocardium, the apoptotic rate in the hBcl-xL-MSC group was significantly lower than those of the medium group and the vector-MSC group (31% ± 1.4% versus 52% ± 1.8% and 42% ± 2.2%, resp., *n* = 6, *P* < 0.001) (Figure 6). These results demonstrated that the hBcl-xL modification could protect MSCs against apoptosis after transplantation into the infarcted myocardium. And the surviving MSCs could surely protect the surrounding infarcted myocardium from further damage, which was favorable for the recovery of heart function.

### 3.7. Bcl-xL Modified MSCs Promoted Angiogenesis and Prevented Scar Formation in Infarcted Heart

**Figure 7:**
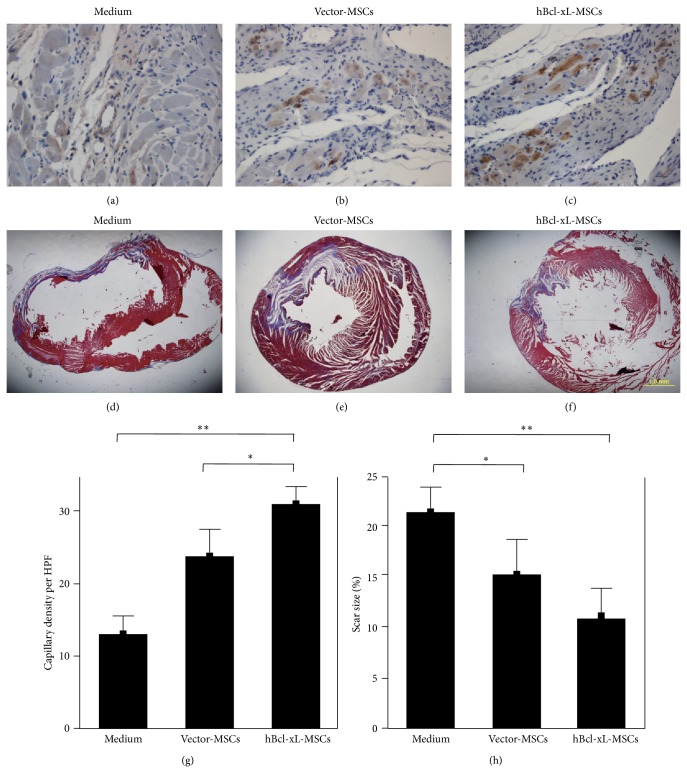
hBcl-xL-MSCs promoted angiogenesis and prevented scar formation in infarcted heart. ((a)–(c)) Evaluation of capillary density by von Willebrand factor staining at 4 weeks after transplantation of medium (a), vector-MSCs (b), or hBcl-xL-MSCs (c). ((d)–(f)) Assessment of scar formation by Trichrome-Masson staining at 4 weeks after medium (d), vector-MSCs (e), or hBcl-xL-MSCs (f) transplantation. (g) Capillary density was presented as the number of vessels per high-power field. (h) Scar size was presented as the percentage of the collagen deposition area to the whole section area of the left ventricle. Values are mean ± SD (*n* = 6, ^∗^ *P* < 0.01, ^∗∗^ *P* < 0.001). Scale bar represents 50 *μ*m (c) and 1 mm (f), respectively.

The above* in vitro* data showed that the hypoxia-induced cytokine secretions from hBcl-xL-MSCs were much higher than those from wild type MSCs and vector-MSCs. Thus, we expected that hBcl-xL genetic modification of MSCs could improve angiogenesis in the ischemic myocardium after MSC transplantation. To evaluate the angiogenic capacity of the hBcl-xL modified MSCs, 4 weeks after MSC transplantation, capillary density was determined in the border zone of the infarcted heart muscle by von Willebrand factor (vWF) staining (Figures 7(a)–7(c)). The results showed that the capillary densities in vector-MSC and hBcl-xL-MSC groups were significantly higher than in the medium group (23.7 ± 1.5 and 31 ± 1.0 versus 13 ± 1.1, vessels per HPF, *n* = 6, *P* < 0.001) (Figure 7(g)). And, also, the capillary density in hBcl-xL-MSC group was 31% higher than in vector-MSC group (*n* = 6, *P* < 0.01). These results demonstrated that the hBcl-xL modification could significantly enhance the angiogenic capability of MSCs* in vivo* and thus effectively promote the revascularization of the ischemic heart muscle.

Scar formation which resulted from transmural myocardial infarction was a consistent outcome of the animal model of LAD ligation. Extensive collagen deposition was the basis for scar formation. In our animal models, 4 weeks after MSC transplantation, heart sections were stained with Trichrome-Masson method to assess scar formation (Figures 7(d)–7(f)). In this method, the collagen fibers were stained in blue color while the cardiac muscle fibers were stained red and the cell nucleus was black-blue. Scar formation was analyzed by the quantitative assessment of collagen deposition (Figure 7(h)). Compared with medium group (21.1% ± 1.1%), both vector-MSC and hBcl-xL-MSC group exhibited smaller scar size (15.1% ± 1.42% and 11.07% ± 1.05%, resp., *n* = 6, *P* < 0.01). And, also, the collagen deposition in hBcl-xL-MSC group was 26.7% lower than in vector-MSC group (*P* < 0.05). The results suggested that hBcl-xL modified MSCs could efficiently prevent scar formation in infarcted heart muscle.

### 3.8. Bcl-xL Modified MSCs Promoted Functional Recovery of Infarcted Heart

To evaluate the extent of heart functional recovery, 4 weeks after MSC transplantation, transthoracic echocardiographic studies were performed on the anesthetized animals (Table 1). Compared with the sham group, contractile function was impaired in all the other three groups after heart infarction. The transplantation of both vector-MSCs and hBcl-xL-MSCs into the infarcted myocardium could improve left ventricular function. However, the level of heart functional restoration in hBcl-xL-MSC group was much higher than in vector-MSC group.

## 4. Discussion

MSCs derived from adult bone marrow have emerged as a promising cell source for the cell therapy of myocardial infarction. However, the low survival rate of the grafted MSCs severely holds back the therapeutic efficacy of cell transplantation aiming at cardiomyocytes restoration. In this study, we demonstrated that hBcl-xL genetic modification could enhance the survival and the biological functions of MSCs both* in vitro* and* in vivo*. Under hypoxic conditions, hBcl-xL modification could protect MSCs against apoptosis and promote the secretions of VEGF, IGF-1, and PDGF. In the LAD ligation animal model, hBcl-xL modification significantly increased the survival rate and the engraftment of MSCs after transplantation. And the transplantation of hBcl-xL-MSCs efficiently prevented scar formation and improved the recovery of heart function after myocardial infarction. These observations were in agreement with the previously published data that the expression of hBcl-xL in rat heart can inhibit the apoptosis of ischemic cardiomyocytes after myocardial infarction and can prolong the cold preservation time period for cardiac transplants [13, 14]. The present study revealed that genetic modification of MSCs with hBcl-xL gene could be an intriguing strategy to improve the viability of MSCs after implantation into the ischemic heart and thereby lead to a better therapeutic outcome for the treatment of myocardial infarction.

The precise underlying mechanisms of the hBcl-xL-MSCs mediated functional recovery of infarcted heart are unclear. It was showed that the* in situ* survival rate of the implanted cells was closely related to the therapeutic efficacy of MSCs [12]. Previous studies demonstrated that the low survival rate of MSCs after transplantation into infarcted hearts was caused by multiple factors including the lack of oxygen, nutrients, and survival factors, the inflammatory reaction, the host immune rejection, the existence of proapoptotic and cytotoxic factors, and the ischemia-reperfusion (I/R) damage which resulted from repeated ischemia [3, 10, 15, 16]. Recently, it was reported that Fas-Fas Ligand (FasL) interactions between host ischemic myocardial cells and implanted MSCs are responsible for activating cell death signaling in implanted MSCs [17]. The inhibition of this interaction could downregulate the expressions of proapoptotic proteins including caspase-8, caspase-3, Bax, and cytochrome-c and improve cell survival and restore heart function [17]. These observations suggested that both Fas-dependent and mitochondria-dependent apoptotic pathways were involved in the death of implanted MSCs in infarcted heart. It was demonstrated that Bcl-xL plays important antiapoptotic roles in both external and internal apoptosis pathways. Bcl-xL can bind with Bax to prevent the release of cytochrome-c from mitochondria and then to prevent the downstream activation of caspases [18]. Also, Fas and tumor necrosis factor receptor induced apoptosis could be inhibited by overexpression of Bcl-xL [19, 20]. These data support a model in which the Bcl-xL modification promotes functional restoration of infarcted hearts by increasing the survival rate of implanted MSCs through the inhibition of Fas- and mitochondria-dependent apoptosis pathways. Further studies need to be performed to address this hypothesis.

As a type of multipotent stem cell, MSCs can be induced to differentiate into various cell lines including cardiomyocytes [3] and endothelial cells [12, 21]. However, several evidences have showed that, instead of the transdifferentiation of MSCs into cardiomyocytes or endothelial cells, the paracrine of survival and angiogenic factors by MSCs played the main role in the cure of heart infarction by MSC transplantation [12, 16, 22]. It was reported that the grafted MSCs could secrete various kinds of growth factors, cytokines, and chemokines, such as VEGF, IGF, PDGF, hepatocyte growth factor (HGF), stromal cell-derived factor-1 (SDF-1), basic fibroblast growth factor (bFGF), and interleukine-1 (IL-1) [12, 23–25]. These factors have been showed to contribute to the functional improvement of infarcted hearts by promoting angiogenesis and cell survival and preventing myocardium remodeling [23, 25–28]. In our study, overexpression of hBcl-xL significantly increased the secretions of VEGF, IGF-1, and PDGF by rat bone marrow MSCs under hypoxic condition. This finding was concordant with the previously reported data that the overexpressed Bcl-2 gene could upregulate the secretion of VEGF in implanted MSCs and improve heart functional recovery after acute myocardial infarction [12]. These data suggested that the cardioprotective effects of hBcl-xL-MSCs could be at least partly attributed to the high level of paracrine factor expressions.

The mechanism underlying the hBcl-xL mediated upregulation of cytokine secretion by MSCs was not clear. Our study and previous data [25] confirmed that, as a major pathological condition in ischemic hearts, hypoxia could enhance the expressions of VEGF and other cytokines in MSCs. It was reported that Bcl-2 overexpression could effectively enhance the stability of hypoxia-induced VEGF mRNA [29]. And treating melanoma cells with a Bcl-2/Bcl-xL bispecific antisense oligonucleotide resulted in a reduction of hypoxia-induced VEGF secretion [30]. These data indicated that Bcl-2 and Bcl-xL play roles in the regulation of hypoxia-induced cytokine secretion.

Hypoxia-induced cytokine secretion was demonstrated to be mediated by a heterodimeric basic helix-loop-helix transcription factor, hypoxia-inducible factor (HIF) [31]. Hypoxia could induce HIF expression by inhibiting its ubiquitination and degradation [32]. HIF plays a pivotal role in responses to ischemia. HIF-1 could upregulate angiogenic cytokine expression through the phosphatidylinositol 3-kinase (PI3K)/mitogen activated protein kinase (MAPK) signaling pathway [33]. In addition, HIF-1 could reverse hypoxia-induced cell apoptosis by upregulating Bcl-2 expression [31]. And HIF-1 directly regulated Bcl-xL transcription by binding to a hypoxia-responsive element (HRE) in the Bcl-xL promoter [34]. On the contrary, Bcl-2 overexpression increased the protein level and the VEGF-promoter binding activity of HIF-1 [29]. Here, we speculated that Bcl-xL works synergistically with HIF by forming a positive feedback loop in the cellular responses to hypoxia. Further studies are needed to clarify this hypothesis.

Taken together, our study confirmed that Bcl-xL genetic modification could enhance the survival and the hypoxia-induced cytokine secretion in engrafted MSCs and thereby promote the functional recovery of infarcted heart. Transplantation of Bcl-xL engineered MSCs may provide an effective approach in the treatment of heart infarction.

## Supplementary Material

Figure 1: The surface marker expression of rat bone marrow MSCs.
The expressions of selected surface markers of rat bone marrow MSCs were analyzed by Flowcytometry. Mouse IgG1 was used as an isotype control (A); in rat bone marrow MSCs, CD29, CD90 and CD44 were highly expressed (B–D) while the markers for hematopoietic stem cells, CD34 and CD45, were not expressed (E and F).

## Figures and Tables

**Table 1:** Echocardiography at 4 weeks after MSC transplantation.

Group	LVDd (mm)	LVDs (mm)	LVEF (%)	LVFS (%)
Sham	4.57 ± 0.03	2.34 ± 0.04	82.95 ± 0.95	48.80 ± 0.10
Medium	6.87 ± 0.03	5.06 ± 0.07	44.79 ± 0.25	26.35 ± 0.35
Vector-MSC	6.66 ± 0.04^∗^	4.81 ± 0.03^∗^	47.22 ± 0.09^∗^	27.78 ± 0.39^∗^
hBcl-xL-MSC	5.99 ± 0.01^∗Δ^	3.89 ± 0.04^∗Δ^	59.60 ± 0.30^∗Δ^	35.06 ± 0.11^∗Δ^

^∗^Differences of statistical significance versus medium group, *n* = 6, *P* < 0.01.

^Δ^Differences of statistical significance versus vector-MSC group, *n* = 6, *P* < 0.01.
