# Supplementary material for: Bcl-xL Genetic Modification Enhanced the Therapeutic Efficacy of Mesenchymal Stem Cell Transplantation in the Treatment of Heart Infarction
Source: Stem Cells Int. 2015 May 5;2015:176409. doi: 10.1155/2015/176409 (PMC4436513; doi:10.1155/2015/176409)
Supplement: Supplementary file 1 — Figure 1: The surface marker expression of rat bone marrow MSCs. The expressions of selected surface markers of rat bone marrow MSCs were analyzed by Flowcytometry. Mouse IgG1 was used as an isotype control (A); in rat bone marrow MSCs, CD29, CD90 and CD44 were highly expressed (B–D) while the markers for hematopoietic stem cells, CD34 and CD45, were not expressed (E and F). [file 176409.f1.pdf]

Supplementary Material

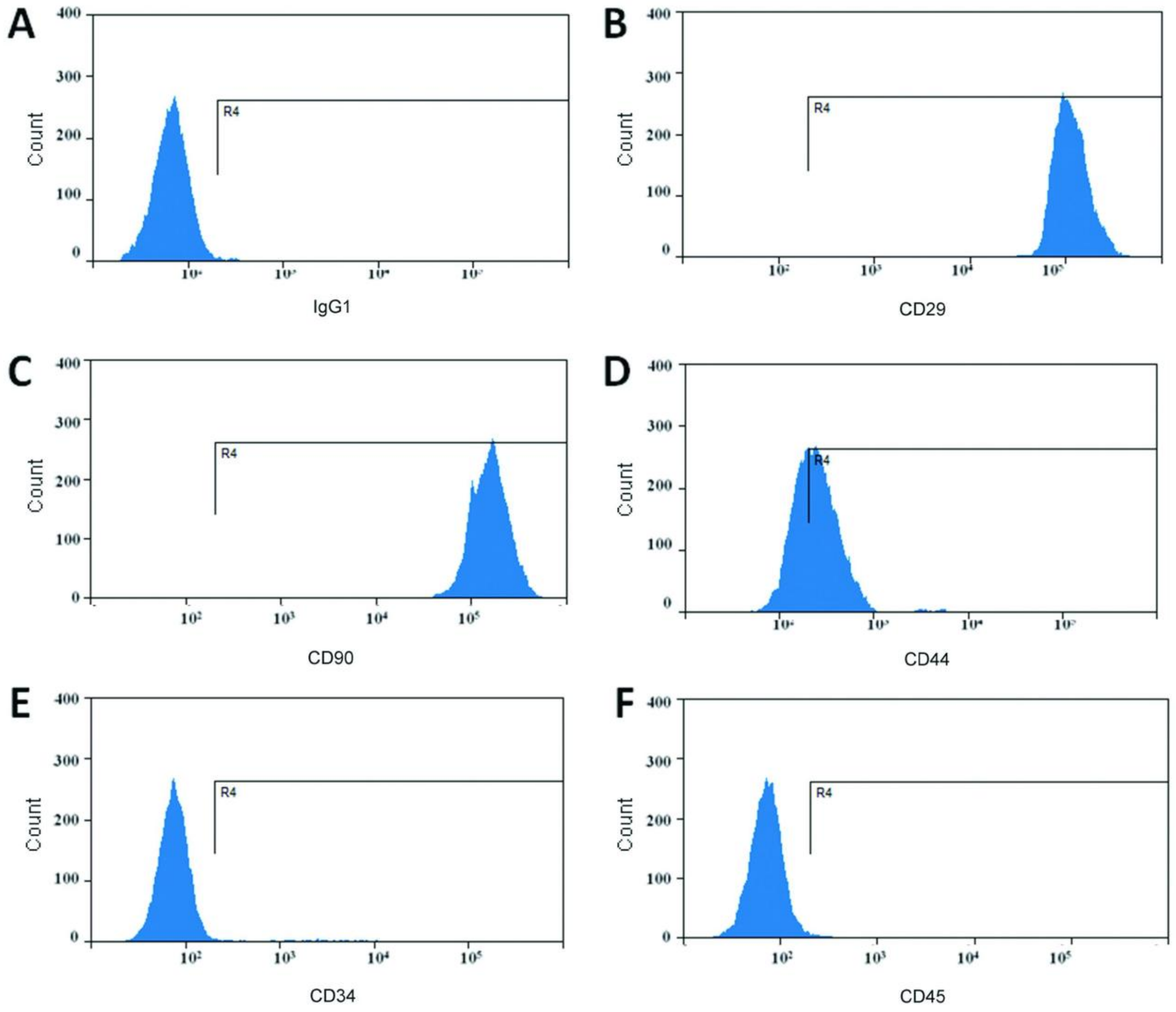

Figure 1, The surface marker expression of rat bone marrow MSCs

The expressions of selected surface markers of rat bone marrow MSCs were analyzed by Flowcytometry. Mouse IgG1 was used as an isotype control (A); in rat bone marrow MSCs, CD29, CD90 and CD44 were highly expressed (B–D) while the markers for hematopoietic stem cells, CD34 and CD45, were not expressed (E and F).
